# Cytotoxic Effects of Hydroxylated Fullerenes in Three Types of Liver Cells

**DOI:** 10.3390/ma6072713

**Published:** 2013-07-09

**Authors:** Kumiko Shimizu, Reiji Kubota, Norihiro Kobayashi, Maiko Tahara, Naoki Sugimoto, Tetsuji Nishimura, Yoshiaki Ikarashi

**Affiliations:** 1 Division of Environmental Chemistry, National Institute of Health Sciences, Kamiyoga 1-18-1, Setagaya-ku, Tokyo 158-8501, Japan; E-Mails: reijik@nihs.go.jp (R.K.); norihiro.kobayashi@nihs.go.jp (N.K.); tahara@nihs.go.jp (M.T.); ikarashi@nihs.go.jp (Y.I.); 2 Division of Food Additives, National Institute of Health Sciences, Kamiyoga 1-18-1, Setagaya-ku, Tokyo 158-8501, Japan; E-Mail: nsugimot@nihs.go.jp; 3 Faculty of Pharmaceutical Sciences, Teikyo Heisei University, Uruidominami 4-1, Ichihara-shi, Chiba 290-0193, Japan; E-Mail: t.nishimura@thu.ac.jp

**Keywords:** hydroxylated fullerene, C_60_, cytotoxic activity, liver cells, mitochondrial damage

## Abstract

Fullerenes C_60_ have attracted considerable attention in the biomedical field due to their interesting properties. Although there has been a concern that C_60_ could be metabolized to hydroxylated fullerenes (C_60_(OH)*_x_*) *in vivo*, there is little information on the effect of hydroxylated C_60_ on liver cells. In the present study, we evaluated the cytotoxic effects of fullerene C_60_ and various hydroxylated C_60_ derivatives, C_60_(OH)_2_, C_60_(OH)_6–12_, C_60_(OH)_12_ and C_60_(OH)_36_, with three different types of liver cells, dRLh-84, HepG2 and primary cultured rat hepatocytes. C_60_, C_60_(OH)_2_ and C_60_(OH)_36_ exhibited little or no cytotoxicity in all of the cell types, while C_60_(OH)_6–12_ and C_60_(OH)_12_ induced cytotoxic effects in dRLh-84 cells, accompanied by the appearance of numerous vacuoles around the nucleus. Moreover, mitochondrial activity in liver cells was significantly inhibited by C_60_(OH)_6–12_ and C_60_(OH)_12_. These results indicate that the number of hydroxyl groups on C_60_(OH)*_x_* contribute to the difference of their cytotoxic potential and mitochondrial damage in liver cells.

## 1. Introduction

**Figure 1:**
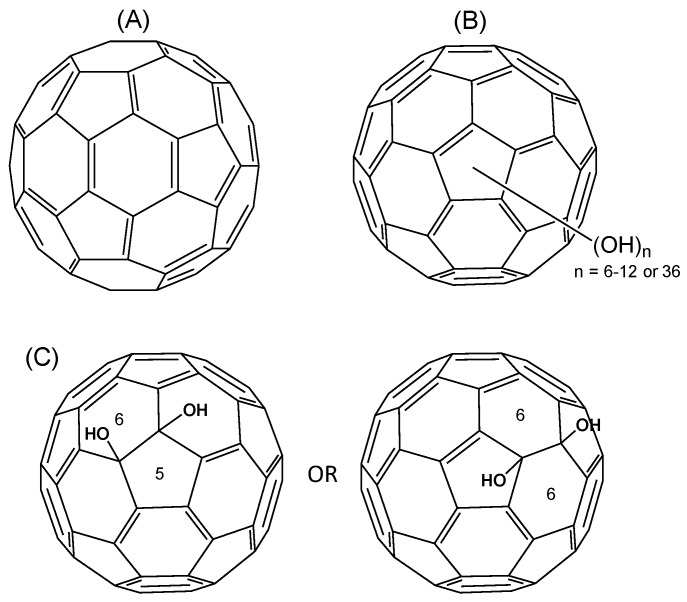
Chemical formulas of C_60_ (**A**); C_60_(OH)_6–12 or 36_ (**B**); and C_60_(OH)_2_ (**C**).

Fullerene C_60_ is comprised of 12 five-membered rings and 20 six-membered rings (Figure 1) [1]. The high chemical stability of fullerenes resists the potential metabolic degradation associated with the carbon cage-opening process under the biological conditions [2,3,4]. Since its discovery in 1985 [5], nanomaterials are applied in various fields due to those useful properties. Fullerene C_60_ and derivatives have anti-cancer and neuroprotective properties as a consequence of antioxidant and free-radical scavenger activity both *in vitro* and *in vivo* [6,7,8,9,10], as well as photo-induced DNA cleavage ability [11]. Moreover, derivatives of fullerene C_60_ have been demonstrated to act as HIV-1 protease inhibitors, which have started to be evaluated in clinical trials [12]. Thus, fullerene C_60_ and its derivatives are expected to have potential applications in the life and medical sciences. However, the insolubility of C_60_ and C_70_ in aqueous solution makes such studies difficult.

Several studies have reported the biodistribution of C_60_ in various experimental animals [13,14,15,16]. We have also reported that the accumulation and decreased concentration of C_60_ in various tissues such as lung, liver, kidney, brain, *etc.*, indicate the possibility of C_60_ and C_60_ metabolites being excreted into feces and/or urine [17]. If it is assumed that C_60_ undergoes *in vivo* enzymatic metabolism in the liver, C_60_ oxidation products such as hydroxylated fullerenes (C_60_(OH)*_x_*) may be produced. At present, the bioavailability of C_60_(OH)*_x_* has begun to garner attention. In human epidermal keratinocyte (HEK) cells, C_60_(OH)_32_ has shown significant cytotoxic activity [18]. Moreover, C_60_(OH)_22–26_ has been shown to induce phototoxicity in human retinal pigment epithelial cells [19]. Yamasaki *et al.* (2006) reported that C_60_(OH)_24_ induced cytotoxicity in human umbilical vein endothelial cells [20]. Nakagawa *et al.* reported that C_60_(OH)_24_ showed cytotoxicity to isolated rat hepatocyte cells [21]. These studies were performed using C_60_(OH)*_x_* containing 22–32 hydroxyl groups, although it is thought that C_60_(OH)*_x_* produced in the metabolic process has a low number of hydroxyl groups. No information has been obtained about the cytotoxic effects of C_60_(OH)*_x_* with a low number of hydroxyl groups in liver cells. Insufficient information is available about the cytotoxicity mechanisms of hydroxylated C_60_ in liver cells. Furthermore, considering the metabolization and adverse effects of anti-cancer drugs and anti-HIV medicines containing fullerene derivatives, the information of the cytotoxic activities of C_60_(OH)*_x_* at the liver is very important.

In the present study, we investigated the cytotoxic effects of C_60_ and the hydroxylated fullerenes, C_60_(OH)_2_, C_60_(OH)_6–12_, C_60_(OH)_12_ and C_60_(OH)_36_ to three types of liver cells, primary cultured rat hepatocytes, dRLh-84 and HepG2. Primary cultured rat hepatocytes maintain phase I, II metabolic activity and uptake transporter activity. dRLh-84 and HepG2 are rat and human hepatoma cells, which have no metabolic activity and were used to evaluate species-differences between rats and humans.

## 2. Results

### 2.1. Mass Spectrometric Analysis of C_60_(OH)_x_

Figure 1 shows chemical formulas of C_60_ and C_60_(OH)*_x_*. The positions which hydroxyl groups of C_60_(OH)_6–12__and 36_ substituted are uncertain. 

**Figure 2:**
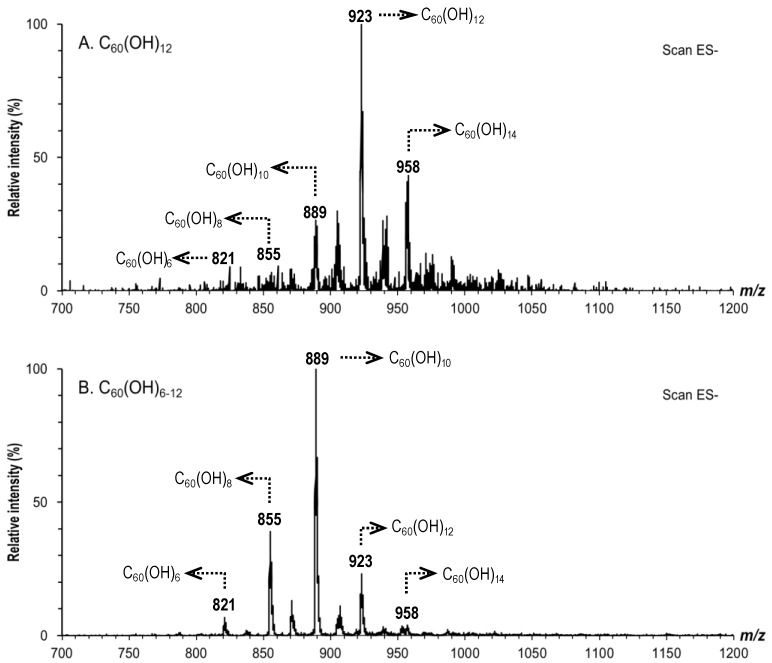
Mass Spectra of C_60_(OH)_12_ and C_60_(OH)_6–12_.

C_60_(OH)_6–12 and 36_ C_60_ and C_60_(OH)_2_ samples showed one signal at *m*/*z* = 720 and *m*/*z* = 754, respectively (data not shown). Five mass spectrometric signals for C_60_(OH)_12_ were observed at *m*/*z* = 821, 855, 889, 923 and 958. The major ion with *m*/*z* = 923 was assigned to C_60_(OH)_12_, and other ions with *m*/*z* = 821, 855, 889, and 958 were assigned to C_60_(OH)_6_, C_60_(OH)_8_, C_60_(OH)_10_, and C_60_(OH)_14_, respectively (Figure 2A). Mass spectrometric signals for C_60_(OH)_6–12_ are shown in Figure 2B. The major ion was assigned to C_60_(OH)_10_, and other ions were assigned to C_60_(OH)_6_, C_60_(OH)_8_, C_60_(OH)_12_ and C_60_(OH)_14_, respectively. 

### 2.2. Cytotoxicity of C_60_ and C_60_(OH)_x_

**Figure 3:**
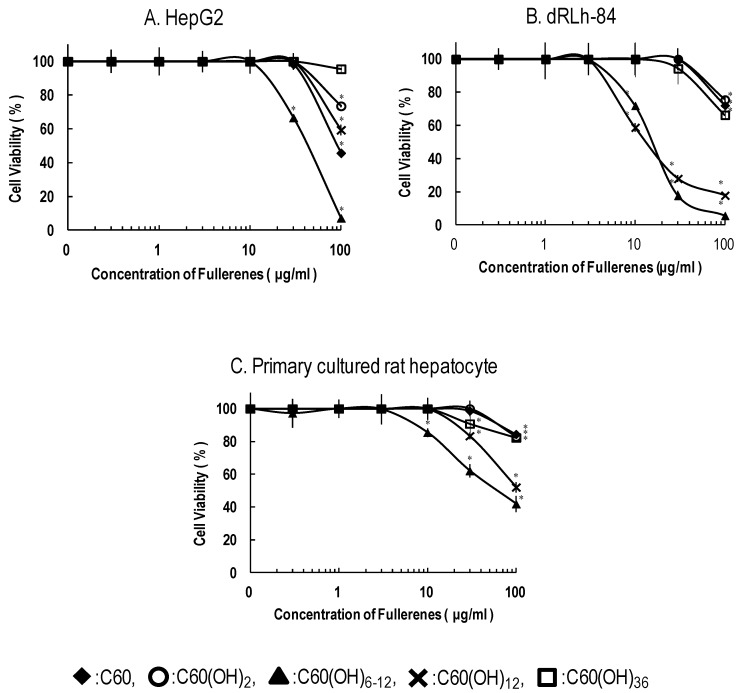
Cytotoxicity of fullerene and hydroxylated fullerenes in liver cells after exposure for 3 days. HepG2 (**A**); dRLh-84 (**B**); and primary cultured rat hepatocytes (**C**) were exposed to C_60_, C_60_(OH)_2_, C_60_(OH)_6–12_, C_60_(OH)_12_, and C_60_(OH)_36_ at concentrations of 0.3–100 μg/mL. After exposure, cytotoxicities were evaluated by the cell viability assay and the values are reported as % viability. Each data represents the mean ± SD (*n* = 3). * Significantly different from the control: *p* < 0.05.

Figure 3 shows cell survival curves of the three types of liver cells exposed to 0–100 μg/mL of C_60_ and C_60_(OH)*_x_*. The maximum concentration was chosen considering suspension’s turbidity and concentrations appeared toxic effects in previous studies [20,21,22]. Among the C_60_(OH)*_x_* tested, C_60_(OH)_6–12_ had the most potent cytotoxic activity. In particular, C_60_(OH)_6–12_ and C_60_(OH)_12_ induced significant toxic activities in dRLh-84 with a dose dependent manner (Figure 3B). Exposure to C_60_(OH)_6–12_ and C_60_(OH)_12_ induced milder cytotoxicity in primary cultured rat hepatocytes than dRLh-84 (Figure 3C). In HepG2, C_60_(OH)_6–12_ showed toxic activity, which was lower than in dRLh-84. On the other hand, C_60_(OH)_12_ had weaker toxic activity in HepG2 than in dRLh-84 (Figure 3A,B). Other C_60_(OH)*_x_* have little or no toxic effects.

**Figure 4:**
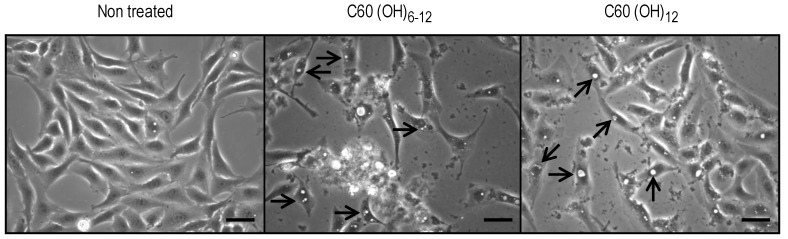
Numerous vacuoles of dRLh-84 cells treated with C_60_(OH)_6–12_ and C_60_(OH)_12_ for 24 h. After dRLh-84 cells were exposed to 30 μg/mL of C_60_(OH)_6–12_ and C_60_(OH)_12_, photographs were taken using an optical microscope. Scale bar: 50 μm. The arrows indicate cytoplasmic vacuoles.

C_60_(OH)_6–12_ and C_60_(OH)_12_ at a concentration of 30 μg/mL caused the formation of numerous vacuoles around the nucleus in dRLh-84 cells (Figure 4). In contrast, the formation of cytoplasmic vacuoles was not detected in HepG2 and primary cultured rat hepatocytes (data not shown).

**Figure 5:**
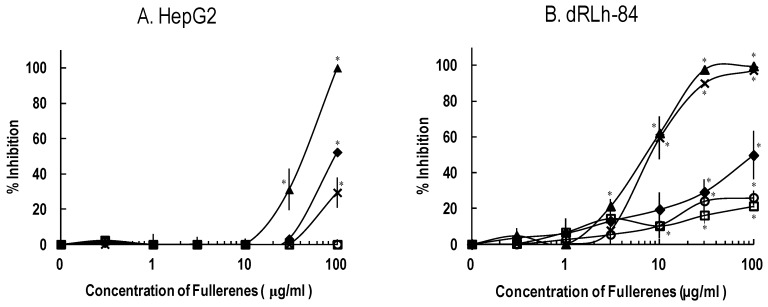

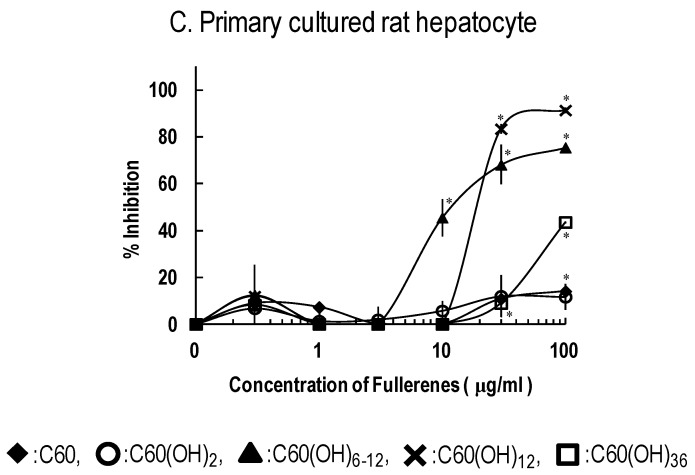
Mitochondrial activity of fullerene and hydroxylated fullerenes in liver cells after exposure for 3 days. Three types of cells, HepG2 (**A**); dRLh-84 (**B**); and primary cultured rat hepatocytes (**C**) were treated with fullerene and hydroxylated fullerenes with the same concentrations as employed in the cell viability assay (the sample symbols are the same as in Figure 3). After exposure for 3 days, the inhibition rate (%) of mitochondrial activity was evaluated. Each data represents the mean ± SD (*n* = 3). * Significantly different from the control: *p* < 0.05.

Mitochondrial succinate-tetrazolium reductase activity in all of the liver cells was inhibited by C_60_(OH)_6–12_ (Figure 5). C_60_(OH)_12_ also inhibited this enzymatic activity in dRLh-84, but provided little inhibition in HepG2 according to cytotoxic activities. The mitochondrial enzyme activity of primary cultured rat hepatocytes was also inhibited by C_60_(OH)_6–12_ and C_60_(OH)_12_ (Figure 5C). Other C_60_(OH)*_x_* had almost a little or no effect.

## 3. Discussion

The molecular diversity of C_60_(OH)*_x_* samples used in this study was analyzed. Some of the molecular diversity of C_60_(OH)_6–12_ and C_60_(OH)_12_ showed overlapping distributions. C_60_(OH)_36_ had many constituents (data not shown). These results indicate that C_60_(OH)*_x_* with the exception of C_60_(OH)_2_ contained various numbers of hydroxyl substituents. Because there is currently no purification technology available for C_60_(OH)_*x*, *x* > 2, *x* < 12_, it was impossible to separate single C_60_(OH)_6, 8, 10_ from C_60_(OH)_6–12_ and C_60_(OH)_12_.

The sensitivities of the three types of liver cells to C_60_(OH)*_x_* differed (Figure 3). C_60_(OH)_6–12_ and C_60_(OH)_12_ exhibited more potent cytotoxic activity in dRLh-84 than in primary cultured rat hepatocytes. Primary cultured rat hepatocytes and dRLh-84 were from the same rat species, but the sensitivities to C_60_(OH)_6–12_ and C_60_(OH)_12_ were different. Thus, it was suspected that metabolic activity may affect the cytotoxicity of C_60_(OH)_6–12_ and C_60_(OH)_12_ in primary cultured rat hepatocytes. 

Meanwhile, C_60_(OH)_6–12_ caused higher cytotoxic activity than C_60_(OH)_12_ in HepG2. In contrast, the cytotoxic activities between C_60_(OH)_6–12_ and C_60_(OH)_12_ show a slight difference. These results indicate that the interspecific difference in hepatoma cells may cause different sensitivities based on number of C_60_(OH)*_x_* hydroxyl groups. Moreover, the malignant grade in the hepatoma might affect the sensitivity to the number of C_60_(OH)*_x_* hydroxyl groups between dRLh-84 and HepG2. 

While there was overlap in the components of C_60_(OH)_6–12_ and C_60_(OH)_12_, C_60_(OH)_6–12_ induced more severe cytotoxic activity than C_60_(OH)_12_ in all of the liver cells. C_60_(OH)_2_ and C_60_(OH)_36_ showed no cytotoxic effects in any liver cells. Although the number of hydroxyl groups that contribute to cytotoxicity cannot be specified, these results suggest that C_60_(OH)_6_, C_60_(OH)_8_, or/and C_60_(OH)_10_ would have more potent cytotoxic activity than other C_60_(OH)*_x_*. For a more detailed understanding, we would need to compare the cytotoxic potential, if it is possible to obtain the purified C_60_(OH)_6_, C_60_(OH)_8_ and C_60_(OH)_10_. Low numbers of C_60_ hydroxyl substituents are expected to be generated by metabolism of C_60_ in the liver after administration. Although the number of hydroxyl substituents produced by hepatic metabolism cannot be identified, if 6–10 hydroxyl substituents were generated from C_60_, cytotoxic activity induced in the hepatoma would be a concern.

Exposure to C_60_(OH)_6–12_ and C_60_(OH)_12_ resulted in the formation of numerous vacuoles around the nucleus in dRLh-84 (Figure 4). Several studies have also reported vacuole formation, or blebbing, upon exposure to C_60_(OH)*_x_* [18,20,21,23]. Yamasaki *et al.* (2006) and Nakagawa *et al.* (2011) have suggested that this morphological change may be caused by depletion of cellular ATP and subsequent autophagosome formation [20,21]. The depletion of protons in the cellular by potent adsorption properties of C_60_ may disturb the ATP synthesis in the mitochondria [24]. Additionally, there have been a few studies of mitochondrial damage caused by C_60_(OH)*_x_* [21,22,25]. In this study, luminescence intensities associated with the ATP content of cells was measured. Low luminescence indicated the depletion of cellular ATP, reflecting functional damage of mitochondria and cell death. Furthermore, the WST-1 assay was used for evaluation of cytotoxicity, which is based on the content of dye produced by mitochondrial enzymes. Therefore, cell death and vacuole formation observed in dRLh-84 exposed to C_60_(OH)_6–12_ and C_60_(OH)_12_ may be caused by damage to mitochondrial functions (Figure 3 and Figure 5). 

Our results are in agreement with previous reports [21,22,25]. In addition, we have shown that C_60_(OH)_6–12_ and C_60_(OH)_12_ effectively caused more mitochondrial damage than other C_60_(OH)*_x_* species in the liver cells. 

## 4. Experimental Section

### 4.1. Chemicals

Fullerene C_60_ (C_60_; nanom purple KN, purity > 99.9%) was purchased from Frontier Carbon Corporation (Fukuoka, Japan). C_60_(OH)_*n*, *n* = 6–12_ (C_60_(OH)_6–12_) was purchased from Kanto Chemical Co. Inc. (Tokyo, Japan). C_60_(OH)_2_ (purity of > 99%), C_60_(OH)_12_ pentahydrate, and C_60_(OH)_36_ octahydrate were purchased from FLOX Corporation (Kanagawa, Japan). With the exception of C_60_(OH)_2_, no information was available on the purity of the C_60_(OH)*_x_* samples. 

Mass spectrometric analysis of C_60_ and C_60_(OH)*_x_* was performed using LC-MS/MS (Waters Alliance 2695 HPLC system—Waters Micromass Quattro Micro API triple quadrupole mass spectrometer, Waters, Milford, USA). 

### 4.2. Sample Preparation

C_60_ was ground in an agate mortar until the color of the powder changed to a brownish-black and was then suspended in dimethyl sulfoxide (DMSO) at a concentration of 10 mg/mL. The stock solution was sonicated and vortexed; and subsequently stored at −20 °C until being used. The C_60_ solution was first diluted with DMSO; and subsequently diluted 100-fold with each growth medium before exposure to cells. C_60_(OH)*_x_* were dissolved in DMSO at a concentration of 10 mg/mL; and were diluted in a manner similar to that used for C_60_. 

### 4.3. Cells

Primary cultured rat hepatocytes and their culture medium were purchased from Biopredic International (Rennes, France). Rat hepatoma cells, dRLh-84, were obtained from the Health Science Research Resources Bank (Osaka, Japan). HepG2 (Human hepatoma cells) were continuously cultured in our laboratory of National Institute of Health Sciences. HepG2 and dRLh-84 were grown in Eagle’s minimum essential medium (MEM, Sigma-Aldrich, MO, USA), supplemented by 10% (v/v) fetal bovine serum (ICN Biochemicals Inc., OH, USA), 50 unit/mL penicillin and 50 μg/mL streptomycin (Gibco, CA, USA), 1 mM sodium pyruvate (Gibco, CA, USA), and 100 μM MEM non-essential amino acid (Gibco, CA, USA). 

### 4.4. Cell Viability Assay

Primary cells were seeded in 96-well plates at 0.3 × 10^6^ cells/mL in 100 μL/well. dRLh-84 cells were seeded at 6 × 10^3^ cells and HepG2 cells were seeded at 2 × 10^4^ cells in 200 μL/well. The cells were incubated overnight at 37 °C in a humidified atmosphere containing 5% CO_2_ in air, and then were exposed to test chemicals. After incubation for 3 days, cell viability was determined using the Cell Titer-Glo Luminescent Cell Viability Assay Kit (Promega, WI, USA). Since the luminescence intensity is based on the ATP content of viable cells, the luminescence intensity of each well was measured using a microplate reader (Mithras LB 940, Berthold technologies, Germany). 

After dRLh-84 were exposed to 30 μg/mL of C_60_(OH)_6–12_ and C_60_(OH)_12_ for 24 h, cells were observed with the optical microscope and photographs of intracellular vacuoles were taken.

### 4.5. Mitochondrial Activity Assay

Mitochondrial activity was measured using the Premix WST-1 kit (Takara, Tokyo, Japan). The absorbance of formazan dye was measured using a microplate reader (Ultraspec Visible Plate Reader II 96, GE Health, Buckinghamshire, UK) at a wavelength of 450 nm, with a reference wavelength of 620 nm. Measured using a microplate reader (Mithras LB 940, Berthold technologies, Germany). 

### 4.6. Statistical Analysis

Statistical analyses were performed using Student’s *t*-test. The test was conducted to verify the difference between each group exposed to fullerenes and the control. Differences with *p* < 0.05 were considered statistical significant.

## 5. Conclusions

C_60_(OH) _6–12_ and C_60_(OH)_12_ showed higher levels of cytotoxicity in liver cells than C_60_, C_60_(OH)_2_, and C_60_(OH)_36_, presumably due to mitochondrial damage. The number of hydroxyl group substituents on C_60_(OH)*_x_* are an important factor in the determination of cytotoxic potential. 
